# Long-term Insights: Histopathological Assessment of Polyurethane Implant Capsules Over 24 Years

**DOI:** 10.1093/asj/sjae057

**Published:** 2024-03-12

**Authors:** Gisela H Pontes, Clara P W Ramos, Lucia de Noronha, Fernando Serra-Guimarães, Amanda S Cavalcanti, Ana Paula F Barbosa, Maria Eugenia L Duarte

## Abstract

**Background:**

Polyurethane (PU)-coated breast implants are known for their strong integration into breast tissue and the formation of capsules around them. However, capsular contracture can pose both aesthetic and clinical challenges.

**Objectives:**

The objectives of this study were to analyze the biological and morphological characteristics of the capsular tissue surrounding PU-coated implants, irrespective of their contracture status, and to assess their potential suitability as a flap in revisional breast surgery for capsular contracture.

**Methods:**

A total of 23 tissue samples were harvested from the capsules surrounding PU-coated breast implants in 12 female patients during replacement or revisional surgery. We evaluated collagen abundance, cellular and vascular density, inflammation, collagen band types and alignment, synovial metaplasia, capsule thickness, and the expression of inflammatory biomarkers and myofibroblasts with immunohistochemical techniques. Scanning electron microscopy was employed to assess implant surface characteristics over time.

**Results:**

We found a significant association of capsule contraction with longer implantation durations and greater implant surface roughness (*P* = .018 and *P* = .033, respectively). Synovial metaplasia was significantly more frequent in noncontracted capsules (*P* = .0049). Both capsule types consisted of paucicellular, type I collagen-rich compact fibrous tissue with low vascularization. There was a marked reduction in inflammatory cells within the foreign body granuloma. The expression of inflammatory biomarkers in the capsular tissue was negligible.

**Conclusions:**

Given the reduced levels of inflammatory and vascular components within the dense, fibrous capsular tissue, we consider them to be viable alternatives for capsular flaps in revisional surgery. This strategy has the potential to mimic the reconstruction achieved with acellular dermal matrix.

**Level of Evidence: 4:**

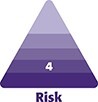

Breast augmentation has become one of the most frequently performed aesthetic surgeries worldwide since the introduction of breast implants into the plastic surgery field in 1962 by Cronin and Gerow.^1^ Since then, these implants have undergone significant advancements and modifications, with the aim of reducing tissue reactions and increasing biocompatibility to enhance their safety and effectiveness.^2^

The initial or first-generation breast implants featured a smooth surface and a thick outer casing and were filled with silicone of moderate viscosity. Additionally, they included a Dacron patch (DuPont, Wilmington, DE) on the ventral side, which helped secure the implant to the chest wall.^2^ However, despite their popularity, these implants were associated with several complications such as silicone gel leakage and migration, calcification of the capsule, and capsular contracture. To address these complications, subsequent generations of implants were developed with modifications aimed at improving biocompatibility, reducing wettability, and lowering the rates of capsular contracture.^3-5^

In 1970, Franklin Ashley pioneered the development of polyurethane (PU) vulcanized coatings for implants.^6^ These PU-coated breast implants boast a uniquely textured surface, distinguishing them from traditional silicone or saline implants. This distinctive surface texture significantly reduces the risk of associated capsular contracture.^7^

Unlike other types of implants, the low rate of contracture associated with PU-coated implants can be partly attributed to the distinct PU degradation process. This degradation involves the activation and mobilization of giant cells and macrophages, which engage in active phagocytosis of PU fragments. This process resembles a granulomatous reaction, similar to that of a foreign body response (FBR). Consequently, thousands of noncoalescing “microcapsules” form around the implant as part of the inflammatory response. As a result, PU-coated implants exhibit strong adhesion to breast tissue and effectively integrate into the capsule that forms around them within the receiving pocket.^8^ Since their introduction, PU-coated implants have been available in several countries. However, there have been a limited number of studies reporting on the biological characteristics of capsular tissue around PU-coated implants.^9,10^

Despite significant advances in various aspects related to improving the characteristics of implant surfaces, the occurrence of both local and systemic complications remains a common concern.^11^ Regardless of the type of implant, 1 prevalent local complication following breast surgery is capsular contracture, with reported prevalence rates ranging from 5% to 19% in cosmetic procedures.^12^ Although the exact cause of capsular contracture is not completely understood, potential contributing factors encompass a complex interplay of immunobiological elements, patient-specific attributes, surgical variables, and implant-specific characteristics.^13^

The choice of treatment for capsular contracture may vary depending on the severity of the condition and the intensity of clinical complaints. The treatment of severe breast capsular contracture (grades III and IV by Baker's classification) involves a surgical procedure. Currently, there is a deficiency in the evidence necessary to ascertain whether capsulectomy surpasses open capsulotomy in its efficacy for mitigating the recurrence of capsular contracture.^14^ However, capsulectomy does not come without risk, and may cause deformities to the breast when striving for total removal of the capsule.^15^

Although PU-coated implants are widely employed in various countries, there has been a lack of studies in which the overall properties of PU-coated implant capsular tissue are investigated. The aim of this study was to assess the biological and morphological properties of contracted and noncontracted capsular tissue around PU-coated implants and evaluate its suitability as a flap in revisional breast surgery for capsular contracture. Additionally, we evaluated implant surface characteristics over time.

## METHODS

**Figure 1.**
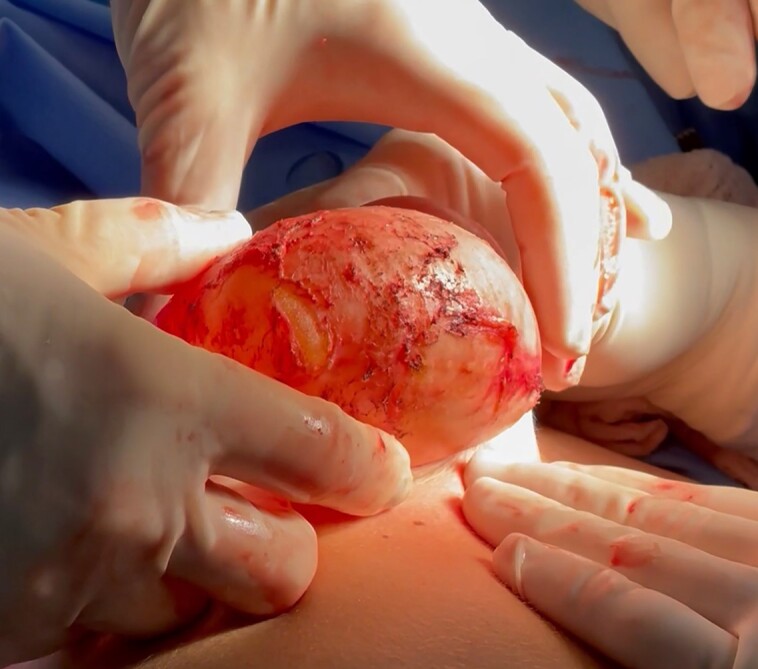
Intraoperative image depicting the explant with the peri-implant capsule during revisional surgery in a 48-year-old female patient.

In this prospective cross-sectional study, we collected a total of 23 representative tissue samples from the periprosthetic tissue (capsule) surrounding PU-coated implants (Silimed, Rio de Janeiro, Brazil) in 12 female patients (ages 44-68 years) during breast implant replacement surgery. The tissue samples were harvested between 6 and 24 years postsurgery, spanning the period from 1998 to 2022. All patients had previously undergone bilateral aesthetic breast surgery, and the implants were originally positioned in the retroglandular space. With the exception of 1 patient, all patients underwent bilateral capsule removal (Figure 1). Before surgery, all patients underwent preoperative evaluations to assess the state of contracture according to Baker's score criteria. Capsules with Baker scores of I and II were considered noncontracted, whereas Baker scores of III and IV were considered contracted. The study protocol was approved by the Rio de Janeiro State University review board and the research was conducted in accordance with the ethical principles outlined in the Declaration of Helsinki. Before participating, each patient provided written consent, by which they agreed to the use and analysis of their data. Clinical trial registration no. 24231219.6.0000.5243.

### Histological Characterization of Capsular Tissue

Capsule tissue samples were formalin-fixed, paraffin-embedded, and subsequently sectioned at 5 μm for hematoxylin and eosin (H&E) and immunohistochemical staining. A semiquantitative approach was utilized to evaluate specific histological parameters within the fibrous tissue of the capsule. These included the abundance of thick, dense collagen fibers, cellularity, vascularization, and the extent of chronic granulomatous reaction FBR. Each of these features was categorized according to 3 levels: 1 for mild or negative, 2 for moderate, and 3 for intense. In addition to these assessments, we also identified the prevailing alignment pattern of collagen bands as either parallel or multidirectional in orientation. Moreover, we observed the presence of synovial metaplasia, characterized by cells arranged in a palisade manner within the innermost portion of the capsule, in direct proximity to the implant. The thickness of each capsule was determined by averaging measurements taken at 5 equidistant points, expressed in micrometers (μm). All histopathological scoring was conducted independently by 2 pathologists who were unaware of the patient's capsular contracture state. This scoring was based on observations from representative H&E-stained capsule samples.

Immunohistochemical analysis was conducted on deparaffinized capsule tissue sections to assess the expression of inflammatory biomarkers and the presence of myofibroblasts. The following primary antibodies were employed: rabbit anti-human α-smooth muscle actin polyclonal antibody (α-SMA, 1:600; Clone: ab5694; Abcam, Cambridge, UK); rabbit anti-human transforming growth factor-beta 1 polyclonal antibody (TGF-β1, 1:200; Clone: E11262; Spring Bioscience, Pleasanton, CA); rabbit anti-human interleukin-4 polyclonal antibody (IL-4, 1:200; Clone: PA5-25165; Thermo Fisher Scientific, Waltham, MA); rabbit anti-human interleukin-13 polyclonal antibody (IL-13, 1:100; Clone: P130-E; Thermo Fisher Scientific, Waltham, MA); rabbit anti-human sphingosine-1 polyclonal antibody (sphingosine-1, 1:200; Clone: Ab71700; Abcam, Cambridge, UK); mouse anti-human CD-44v6 monoclonal antibody (CD-44, 1:200; Clone: VFF-7; Novocastra Laboratories, Newcastle upon Tyne, UK), and rabbit anti-human MMP-9 monoclonal antibody (MMP-9, 1:200; Clone: EP1254; Abcam). Immunohistochemical reactions were developed with the Mouse and Rabbit Specific HRP/DAB IHC Detection Kit—Micropolymer (Abcam, ab236466, Cambridge, UK) and lightly counterstained with Harris hematoxylin for visualization.

The immunohistochemical reactions were assessed with a slide scanner (Axio Scan.Z1; Zeiss, Oberkochen, Germany). Digital images of high-magnification fields (HPFs) were acquired in tagged image file format (TIFF) and subsequently quantified employing Image-Pro Plus software (Media Cybernetics, Rockville, MD). At least 10 HPFs of each slide were analyzed, with cells exhibiting brown cytoplasmic immunostaining identified as positive. For each primary antibody, a mean immunopositive area percentage per HPF was computed for every sample. All percentage-based results were automatically imported into a spreadsheet for statistical analysis. Similarly, the percentages of type I and type III collagen content within the fibrous capsule were determined from sirius red–stained slides with the same imaging system and methodology as described for immunohistochemistry.

### Implant Surface Characterization

To characterize the surface of the PU-coated implants, we utilized scanning electron microscopy (SEM) with a spatial resolution of 30 nm. The SEM system was equipped with a fixed acceleration voltage of 15 kV and a conductive high-sensitivity detector for backscattered electrons (HITACHI TM-1000; Hitachi, Tokyo, Japan). The average surface roughness (Ra), a measure of microirregularities on the implant surface, was determined. Ra values were expressed as median and standard deviation.

### Statistical Analysis

To analyze the data, the normality of the continuous variables was assessed with the D’Agostino and Pearson and Shapiro–Wilk tests. If the data followed a normal distribution, parametric tests were employed. Nonparametric methods were applied for group comparisons when the data did not follow a normal distribution. For the comparison of semiquantitative histological variables between contracted and uncontracted capsules, we utilized the chi-square test or Fisher's exact test. The Mann–Whitney test was employed to analyze the capsule thickness (median ± standard error) and the percentage of immunopositive staining by Baker's score. Further pairwise comparisons were conducted with an unpaired *t* test with Prism 8.2.1 (GraphPad Software, La Jolla, CA). Statistical significance was determined by *P* values less than .05.

## RESULTS

### Capsule Tissue Architecture and Morphology

**Figure 2.**
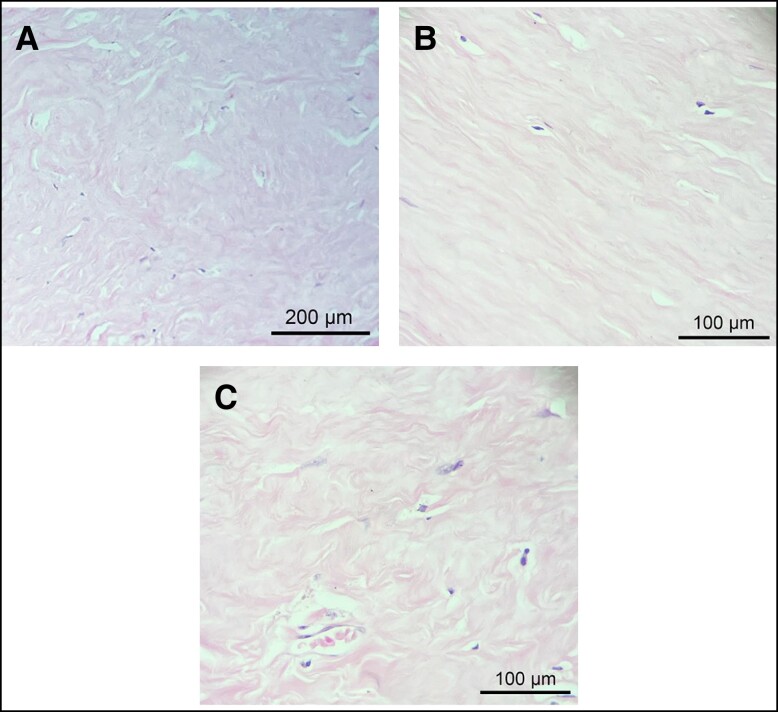
Histopathological findings of the capsular connective tissue. (A) Thick, dense bands of type I collagen without any particular alignment pattern. (B) Low cell density throughout the connective tissue of the capsule. (C) Poor vascularity of the dense connective tissue of the capsule. Representative images of hematoxylin and eosin staining of contracted (A and C) and uncontracted (B) capsules.

The examination of capsule tissue architecture and morphology revealed no significant difference in type I collagen fiber density or alignment between contracted capsules (*n* = 16) and uncontracted capsules (*n* = 7) (Table 1). In both cases, the capsules consisted of densely packed bands of compact type I collagen, distributed over the entire thickness of the capsule, without exhibiting any particular alignment pattern (Figure 2A). Throughout the fibrous tissue of the capsule, fibers displayed both parallel and multidirectional orientations. Similarly, no significant difference in terms of cellularity or vascularization was observed between the 2 capsule types, regardless of their contracture state (Table 1). Both contracted and uncontracted capsules were paucicellular, with cells sparsely distributed within the connective tissue of the capsule (Figure 2B). Furthermore, the compact fibrous tissue showed either no or significantly reduced vascularization, as shown in Figure 2C.

**Table 1.** Comparative Analysis of Connective Tissue Morphology in Contracted and Uncontracted Capsules

Histological variable	Contracted Uncontracted		*P* value
Type I collagen fiber density	1 (0/16)	1 (0/7)	.80
	2 (10/16)	2 (4/7)	
	3 (6/16)	3 (3/7)	
Type I collagen fiber alignment	P (6/16)	P (1/7)	.99
	M (10/16)	M (6/7)	
Overall cellularity	1 (10/16)	1 (4/7)	.99
	2 (6/16)	2 (3/7)	
	3 (0/16)	3 (0/7)	
Tissue vascularization	1 (10/16)	1 (5/7)	.99
	2 (6/16)	2 (2/7)	
	3 (0/16)	3 (0/7)	
Chronic foreign body reaction	1 (5/16)	1 (2/7)	.69
	2 (6/16)	2 (2/7)	
	3 (5/16)	3 (3/7)	
Synovial metaplasia	Yes (3/16)	Yes (6/7)	.0049 ^a^
	No (13/16)	No (1/7)	

^a^Synovial metaplasia was significantly more frequent in uncontracted capsules than in contracted capsules. Histological findings were semiquantitatively graded on a scale: grade 1 signifies mild or absent findings; grade 2 represents a moderate level of findings; and grade 3 indicates intense findings. Collagen band alignment was denoted as “P” for parallel alignment and “M” for multidirectional alignment. The presence of synovial metaplasia is indicated as “Yes,” while its absence is indicated as “No.”

**Figure 3.**
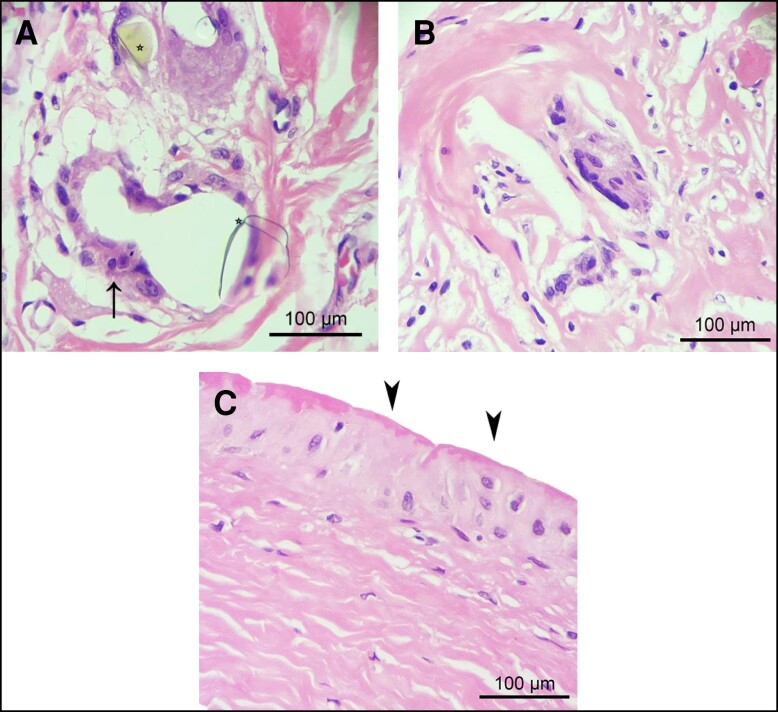
Chronic foreign body reaction and synovial metaplasia in contracted and uncontracted capsules. (A) Within the connective tissue of both types of capsules, a prevalent chronic foreign body reaction characterized by the presence of polynucleate foreign body giant cells (indicated by arrow) in association with refringent crystalline amorphous material was observed. This crystalline amorphous material is likely derived from polyurethane foam–coated implants. (B) Chronic foreign body reaction exhibiting a reduced inflammatory cell response. (C) Synovial metaplasia (arrowheads), characterized by the proliferation of synovial-like cells arranged in a palisade manner in the innermost region of the capsule directly interfacing with the polyurethane implant, was significantly more frequent in uncontracted capsules than in contracted capsules (*P* < .0049). Representative images of hematoxylin and eosin staining of contracted (A) and uncontracted (B and C) capsules.

A chronic FBR was commonly observed in the capsular tissue, with similar intensity observed in both types of capsules. In particular, the presence of polynucleate foreign body giant cells (FBGCs) associated with refringent crystalline amorphous material (Figure 3A), believed to originate from the PU-coated implants, was only sporadically accompanied by a small amount of lymphohistiocytic infiltrate (Figure 3B). Nevertheless, in general, the FBR exhibited a notable depletion of inflammatory cells. Furthermore, the incidence of synovial metaplasia was significantly higher in uncontracted capsules than in contracted capsules (Table 1 and Figure 3C). No cases of anaplastic large cell lymphoma were observed in the studied sample.

**Figure 4.**
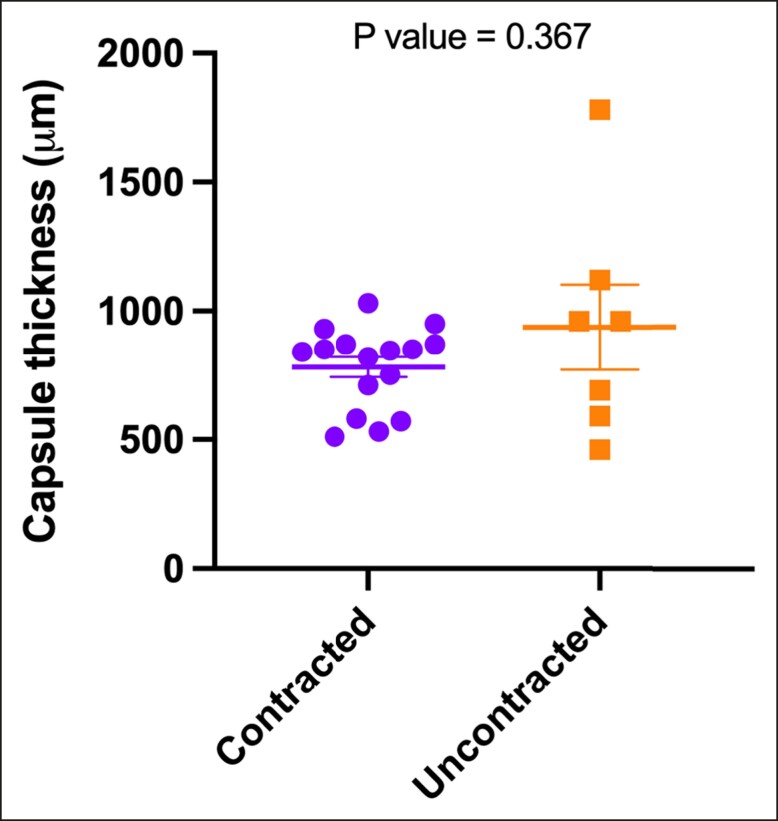
The median values of 5 distinct thickness measurements in micrometers (μm) were determined from hematoxylin and eosin–stained tissue sections obtained from both contracted and uncontracted capsules. The represented data are depicted as the median and standard deviation. Statistical analysis with the Mann–Whitney test yielded a *P* value of .367, indicating no significant difference between the 2 groups.

There was no statistically significant difference in capsule thickness between the contracted group (median 843 μm ± 39.5 μm, range 510-1030 μm) and the uncontracted group (median 960 μm ± 166 μm, range 460-1780 μm) (Figure 4).

### Implant Age and Surface

**Figure 5.**
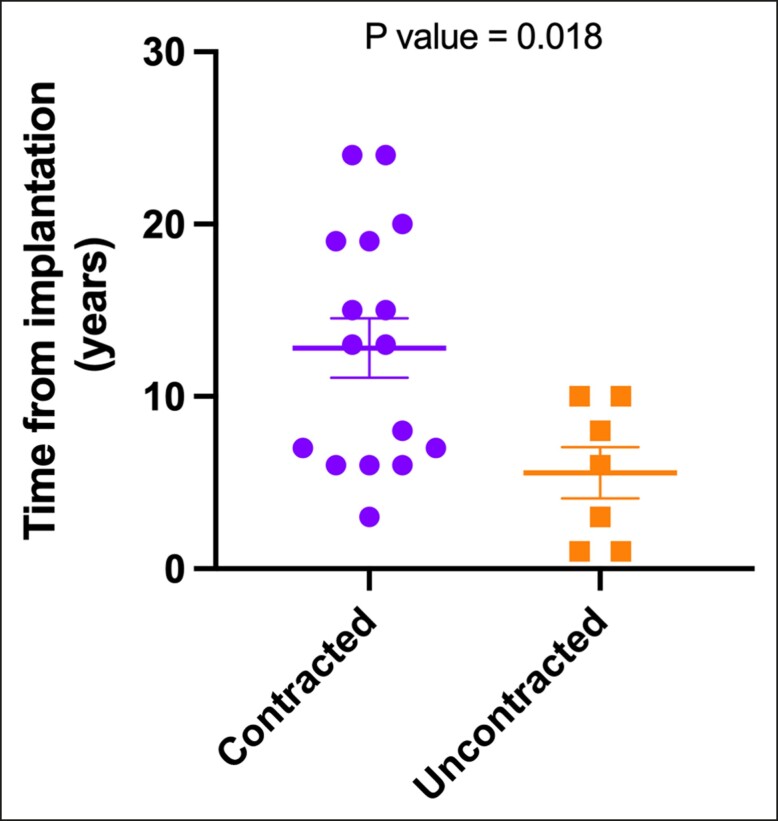
Contracture status and implant age. Statistical analysis with the unpaired *t* test yielded a *P* value of .018, indicating an association between capsule contraction and longer implantation durations.

**Figure 6.**
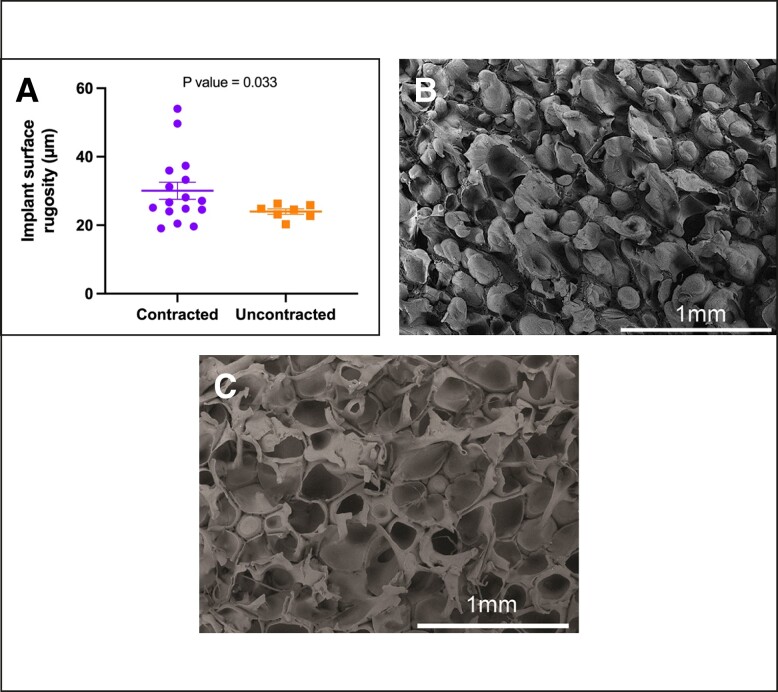
Contracture status and the surface roughness (Ra) of polyurethane foam–coated implants (Silimed). Surface roughness refers to the measurement of microirregularities found on the implant surface, as observed under scanning electron microscopy. (A) The roughness of the implant surface is significantly higher in contracted capsules (*P* = .033). (B) Detailed views of the surface characteristics of contracted capsules and (C) uncontracted capsules.

**Figure 7.**
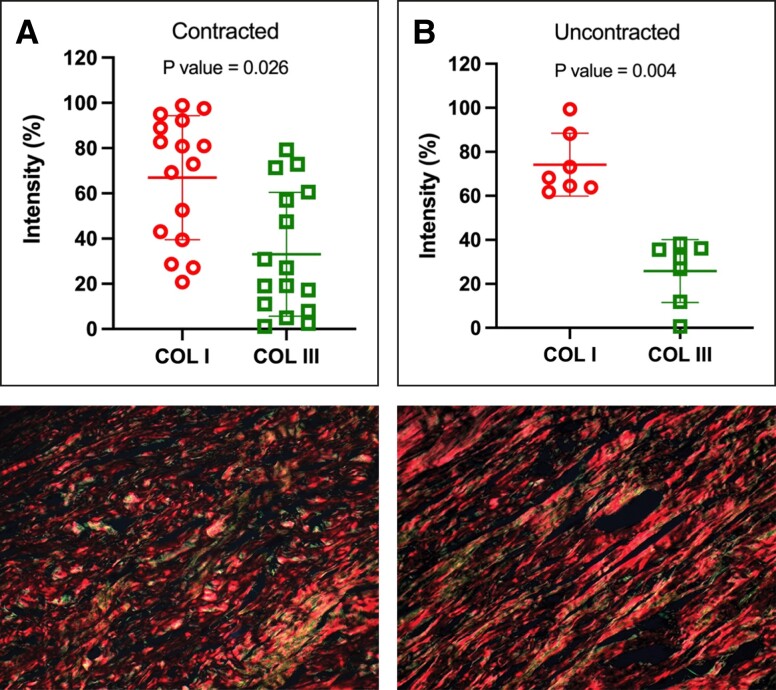
Abundance of type I and type III collagen within the capsules. Both contracted (A) and uncontracted (B) capsules exhibited a higher proportion of type I collagen than type III collagen. (C) Representative histologic sections, stained with picrosirius red and examined under polarized light microscopy, of a contracted and (D) uncontracted capsule. P values were calculated with a paired *t* test.

Regarding implant age, we observed a significant association (*P* = .018) between contracted capsules and longer implantation durations. The median implantation time for contracted capsules was 13 years ± 1.73 years (range 3-24 years), in contrast to uncontracted capsules, with a median of 6 years ± 1.49 years (range 1-10 years) (Figure 5). When assessing implant surface roughness with SEM, we identified a significant difference (*P* = .033) between the 2 types of capsules (Figure 6A). Contracted capsules exhibited greater surface roughness, with a median of 26.9 μm ± 2.52 μm (range 19.1-54 μm), while uncontracted capsules displayed a median roughness of 24.6 μm ± 0.79 μm (range 20.3-26.3 μm) (Figure 6B, C). Despite the presence of contracture, our analysis with sirius red staining revealed that the proportion of type I collagen was significantly higher than that of type III collagen in both contracted (66.9% ± 27.4% vs 33.1% ± 27.4, *P* = .026) and uncontracted capsules (74.2% ± 14.3% vs 25.9± 14.3, *P* = .004) (Figure 7).

### Immunohistopathological Findings

No statistically significant differences were observed in the levels of inflammatory biomarkers (IL-4, IL-13, TGF-β, Sphing), CD-44, MMP-9. or in α-SMA immunoreactivity based on contracture status. Overall, these biomarkers were found to be only negligibly expressed in the fibrous capsules, as summarized in Table 2.

**Table 2.** Comparison of Expression of Inflammatory Biomarkers and Myofibroblast Markers Between Tissues of Contracted and Uncontracted Capsules

Biomarker	Contracted^a^	Uncontracted^a^	*P* value
IL-4	10.61 (2.46-16.25)	7.06 (2.34-12.67)	.147
IL-13	1.07 (0.12-7.89)	0.34 (0.06-3.94)	.367
Sphing	2.33 (0.10-11.70)	2.08 (0.17-5.14)	.526
TGF-β1	1.53 (0.05-4.36)	0.86 (0.03-6.58)	.664
α-SMA	2.61 (0.54-7.80)	1.42 (0.27-16.74)	.815

Tissue expression presented as percentage per high-magnification field. ^a^Median (minimum–maximum) values. *P* values were calculated with the nonparametric Mann–Whitney test.

## DISCUSSION

While there is an inherent risk of developing capsular contracture, breast augmentation and reconstruction with silicone implants rank among the most commonly performed cosmetic surgeries worldwide. In this study, we conducted a prospective cross-sectional analysis involving a total of 23 tissue samples harvested between 6 and 24 years following surgery from the capsular tissue surrounding PU-coated breast implants from 12 patients during replacement or revisional surgery. Our specific focus was on understanding the biological and morphological properties of the capsule tissue that forms around PU-coated implants.

Despite the increasing application of PU-coated implants in reconstruction and augmentation, there is a lack of relevant information, including major biological findings of the capsule surrounding PU-coated implants.^10,16,17^ Regardless of whether capsular contracture has occurred, we considered assessment of the structural and biological characteristics of the tissue surrounding PU-coated implants the first step in investigating and determining its viability as a potential capsular flap in secondary surgery.

Extensive research has been conducted concerning the capsular tissue formation around breast implants. The scarring process surrounding the implant is intricately linked to the implant's surface characteristics as it interacts with breast tissue.^18,19^ In the specific case of PU-coated breast implants, giant cells that develop as part of the inflammatory process are unable to break down the PU residue. Consequently, this particulate material becomes integrated into the capsule tissue for up to 30 years after the primary surgery, effectively increasing its thickness.^20^ This phenomenon results in the formation of small capsules that remain distinct and do not merge together, thereby associating this type of breast implant surface with the lowest rates of capsular contracture.^8,21^ Indeed, an observational study of 300 patients submitted to primary breast augmentation with PU-coated implants and followed up for 10 to 18 years reported a contracture rate of 0.7%.^22^ In a systematic review, the reported rate of capsular contracture in primary reconstructive surgeries with PU-coated implants ranged from 1.8 to 3.4%, while in primary augmentation procedures, the capsular contracture rate ranged from 0.4% to 1% for the same type of implant.^17^

As confirmed by previous investigators of breast implants in general, we have observed an increasing likelihood of capsule contraction over time in PU-coated implants.^9,20,23,24^ In our series, all samples obtained from noncontracted capsules were harvested from patients less than 10 years postoperatively, in contrast to contracted capsules, which were observed in 70% of patients within 13 to 24 years after their initial breast surgery. A retrospective analysis revealed reported cases of early-onset capsular contracture, occurring within 3 years after breast reconstruction surgery, when PU-coated implants were associated with radiotherapy.^7^

Based on SEM imaging analysis, we observed a positive association between capsule contraction and higher average implant surface roughness. In an experimental model, it has been demonstrated that tissue ingrowth on the implant's surface is closely linked to the complexity of its textures.^25^ The gradual increase in the surface roughness of the implant over time could influence tissue responses and healing processes and account for the elevated rate of contracture, because there would be reduced incorporation of PU into the capsule. This increased permeation of fibrous tissue into the implant's surface has been shown to correlate with lower rates of synovial metaplasia, likely due to reduced movement between the implant and the surrounding tissue.^25^ In fact, our results unequivocally demonstrate increased rates of synovial metaplasia in noncontracted capsules.

The exact mechanism underlying the dynamics of connective tissue remodeling in capsules related to PU-coated implants over time is not fully understood. In our examination, the capsules shared some common histological characteristics, irrespective of their contraction status. They predominantly consisted of poorly vascularized, paucicellular tissue rich in type I collagen, with no discernible alignment pattern of collagen bands or striking structural differences in collagen distribution throughout the thickness of the capsules. Similar findings were reported in a study that involved correlating patients’ clinical data with histological parameters after the explantation of both smooth and textured devices (excluding PU-coated implants). According to the results, the majority of the tissue consisted of uniformly distributed collagen fibers.^26^ Furthermore, a wide variety of capsule architectures, emphasizing the generally low cellularity and the prevalence of thick, aligned type I collagen bands within contracted capsules, have already been documented.^27^

In contrast to other authors we were unable to establish a relationship between the capsule thickness and any other clinical or morphological finding.^28^ Notably, the content of type I collagen within the capsules was found to be 2 and 3 times greater than the content of type III collagen in contracted and noncontracted capsules, respectively. Our findings align with those observed in an experimental rabbit model, indicating that capsules around PU-coated implants have less fibrotic tissue and type III collagen than those around textured implants.^21^

In a comprehensive review, Shin et al described in detail the overall FBR from the initial inflammation stage to fibrotic capsule formation after the insertion of a foreign body, such as breast implants.^29^ During the early stages of the inflammatory process, lymphocytes and monocytes locally secrete several growth factors and cytokines.^30^ These bioactive molecules activate macrophages and induce their fusion into FBGCs. Activated FBGCs, along with fibroblasts and myofibroblasts, play pivotal roles in establishing and stabilizing the biological reactions that ultimately result in capsular fibrosis.^29^

The histopathological findings from explanted breast implant capsules were described in a previous retrospective study.^31^ Despite the absence of information regarding the surface type of the implants, the authors described the primary finding within the capsules as compact fibrous tissue characterized by collagen fibers displaying an inconsistent and disorganized overall pattern. The inflammatory response within the fibrous tissue was marked by occasional mononuclear cells, with some foci containing lymphocytes, histiocytes, and FBGCs.^31^ In our examination, we observed that the FBR was quite common in both capsule types, with FBGCs containing intracytoplasmic refringent crystalline material, which was assumed to originate from the PU coating of the implants. We consider the scarcity of inflammatory cellular participation in granuloma formation and maintenance to be a significant finding from our current study. Furthermore, this finding was consistent with the minimal expression of inflammatory biomarkers we observed in our quantitative immunohistochemical analyses.

Recent studies have focused on the systemic inflammatory reaction to silicone implants and have shown an association with diseases of chronic inflammation such as breast implant–associated anaplastic large cell lymphoma (BIA-ALCL).^32^ The mechanism involved in the development of lymphomas may involve an immune response induced by the silicone or polyurethane material of the implant, which can cause an exaggerated immunological reaction and induce monoclonal neoplasia with activated T lymphocytes.^33^ Other postulated mechanisms involve an indirect reaction mediated by cytokines and silicone-induced toxic damage.^34,35^ Repeated exposure to antigens leading to chronic inflammation has been demonstrated to result in prolonged activation and recruitment of T cells, a phenomenon often linked with T-cell lymphomas. The process of lymphomagenesis is intricately connected to genetic instability within the inflammatory microenvironment, which facilitates the emergence of malignant clonal T-cell populations. This is followed by an increase in the expression of cytokines that promote tumor growth, including interleukin (IL)-6, IL-10, and IL-17.^34,36^ Kadin et al further confirmed the presence of a Th17/Th1 phenotype in BIA-ALCL tumor lymphocytes, providing additional evidence for the potential involvement of antigenic stimulation and chronic inflammation in the initiation and promotion of BIA-ALCL.^36^

Regarding the characteristics of this understudied tissue, this research can serve as a foundation for future investigations aimed at assessing its interactions with local tissues and the outcomes associated with PU-coated implants. Capsular tissue that forms around breast implants can serve as a versatile and safe resource, with wide applicability in various secondary breast surgeries, including implant replacement on the retromuscular plane and breast reconstruction for lateral implant stabilization. A dermocapsular flap technique, which elevates the inframammary fold, corrects breast ptosis, covers implant exposure, and addresses implant contour deformities, has already been described in previous research.^37^

Another approach introduces 2 techniques: 1 for covering the lower pole of the breast, correcting the inframammary fold, and the other for stabilizing it. This approach offers a simple, stable, and reproducible solution to prevent lateral implant displacement.^38^ An alternative option involves applying acellular dermal matrix (ADM) to cover the lower breast pole; however, this approach may increase procedure costs and is not always feasible due to material availability constraints.^39^ These methods offer straightforward, dependable, and replicable solutions, effectively mitigating the risk of implant migration.

Based on our findings, we suggest that the tissue that develops around PU-coated breast implants can serve as a versatile and secure resource with broad applications in various surgical scenarios. It can be effectively utilized in revisional surgeries, such as implant exchanges with retromuscular plane placement, as well as in breast reconstructions to enhance lateral implant stability. Furthermore, this tissue can serve as the foundation for a dermocapsular flap, similar to ADMs, which can elevate the inframammary fold, correct breast ptosis, provide coverage for exposed implants, and address implant contour deformities.^37^

One limitation of this study was the lack of further investigations into the molecular mechanisms that regulate the macrophage response and FBR at the interface between tissue and the breast implant, as well as how the cross-talk between local cells affects biomaterial surface properties. A further limitation of our study was the failure to investigate the presence of biofilm in the capsule samples, as well as its potential clinical implications for their utilization in revisional surgeries. Further studies are required to gain a comprehensive understanding of the multifactorial fibrotic process that develops around PU-coated breast implants, confirming the versatility of this tissue for revisional surgeries.

## CONCLUSIONS

Our results emphasize several aspects of the fibrous capsule that forms around PU-coated breast implants, irrespective of whether the capsule is contracted or uncontracted. The capsule comprises paucicellular tissue with limited vascularization and is rich in type I collagen, indicating its mature scar tissue nature. The diminished presence of inflammatory and vascular components within the capsule theoretically renders this tissue a promising and viable candidate for off-the-shelf use as a capsular flap in revisional surgery, potentially replicating the reconstruction achieved with ADM.
